# Dynamics of coffee leaf rust (*Hemileia vastatrix*) in Puerto Rico: associations with season, cultivar, altitude, and hyperparasites

**DOI:** 10.3389/fpls.2026.1768440

**Published:** 2026-03-10

**Authors:** Yobana A. Mariño, Diana P. Buitrago, Luz M. Serrato-Díaz, Emily Almonte-Javier, Sebastián E. Negrón Moreno, Paul Bayman

**Affiliations:** 1 Department of Biology, University of Puerto Rico – Río Piedras, San Juan, PR, United States; 2 USDA-ARS-Tropical Agriculture Research Station (TARS), Mayagüez, PR, United States

**Keywords:** coffee cultivars, coffee leaf rust, hyperparasite, incidence, resistance, temperature

## Abstract

Coffee leaf rust (CLR), caused by the fungus *Hemileia vastatrix*, is the most serious disease of coffee worldwide. CLR has been present in Puerto Rico since 1989, yet its incidence and severity have not been reported. We surveyed CLR incidence and severity for three years in fifteen sites and tested associations with environmental variables. CLR hyperparasites were also surveyed. CLR incidence ranged from 0% to 100% and severity from 0% to 60%. Both incidence and severity increased during the dry season following harvest. Environmental variables were important drivers for CLR and its hyperparasites in the field. The incidence and severity of both CLR and its hyperparasites were negatively correlated with temperature and rainfall. While CLR was positively correlated with mean relative humidity and leaf wetness, hyperparasites showed a negative correlation with both minimum and maximum relative humidity. CLR-susceptible cultivars had significantly more CLR than resistant cultivars, but the resistant cultivars also had CLR damage and followed the same seasonal pattern. The resistant cultivar with the most CLR damage was Marsellesa, widely planted to replace CLR-susceptible cultivars lost to Hurricanes Irma and Maria in 2017. CLR incidence and severity both increased significantly with altitude (range 400–951 m). Incidence and severity of hyperparasites followed the same seasonal pattern as CLR. Some of the patterns reported here agree with previous studies in other areas. They form a baseline for management decisions on cultivars and control, and a point of comparison for future studies.

## 1 Introduction

Coffee (*Coffea arabica* L.) is a beloved global commodity and the most widely traded tropical crop (FAO, 2025). Coffee leaf rust (CLR), caused by the fungus *Hemileia vastatrix* (Berk. & Broome), is the most destructive disease of the coffee plant and a significant limiting factor in its production (Avelino et al., 2015). This disease can reduce coffee quality and yield by up to 75-80% (Koutouleas, 2023), resulting in annual economic losses of over US$1,000,000,000 (McCook, 2006). A CLR epiphytotic in Central America in 2012–2013 resulted in estimated losses of $500M and over 370,000 job losses (ICO, 2013). CLR is a textbook example of how a plant disease can affect history, economy, and society (Large, 1940).

**Figure 1:**
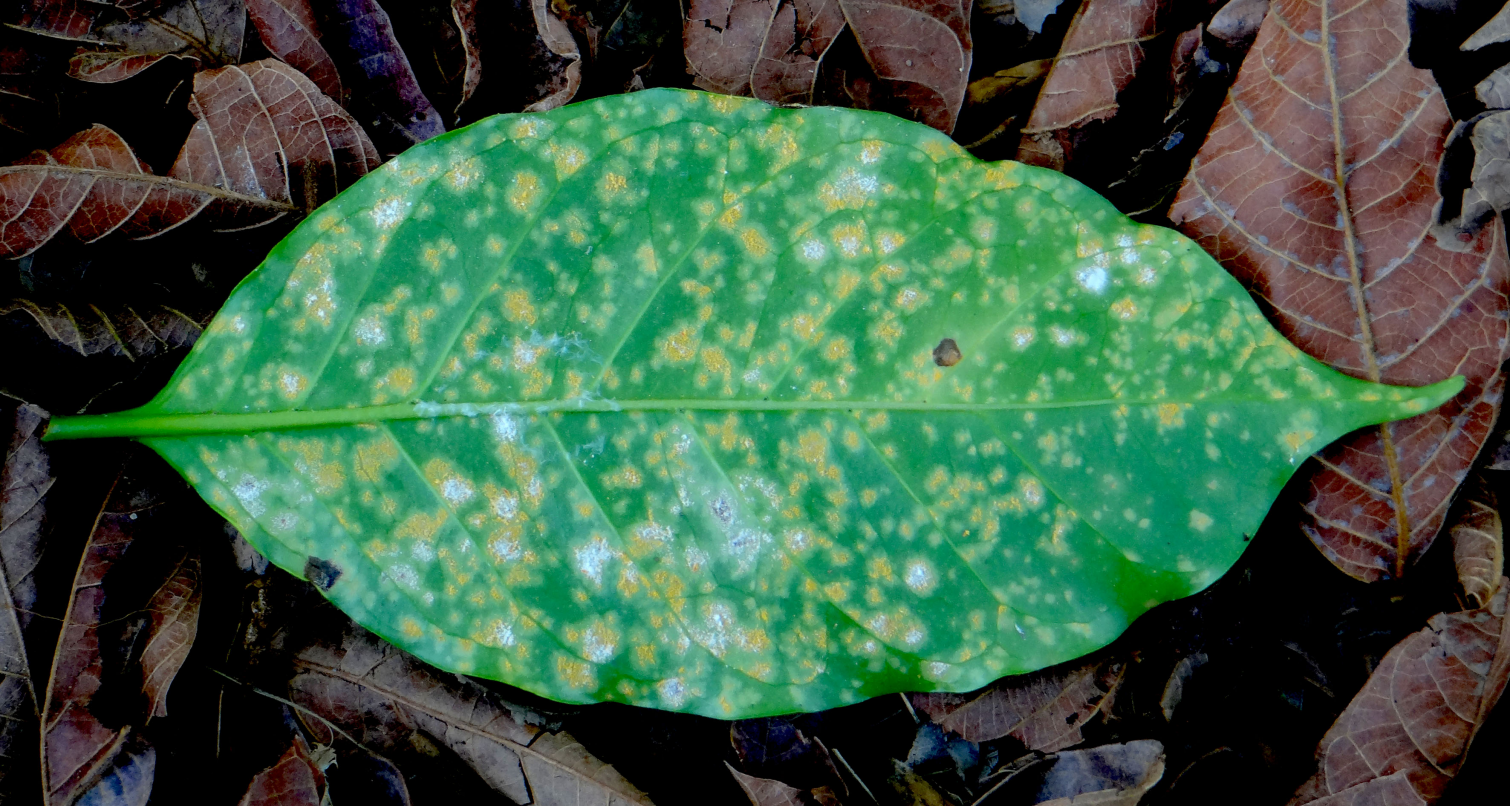
Coffee leaf rust (CLR) lesions caused by the fungus *Hemileia vastatrix* (Berk. & Broome). The disease is characterized by pustules with powdery, orange urediniospores on the lower surface of infected leaves. Hyperparasites, visible as white mycelium, are growing over several CLR pustules.

*H. vastatrix* is a host-specific and biotrophic pathogen (Wellman and Echandi, 1981). Its highly mobile urediniospores can be dispersed by wind, splashing water, insects, and transport of soil, clothing, and equipment. Pustules with powdery, orange urediniospores appear on the lower surface of infected leaves (**Figure 1**) (Gaitán et al., 2015). As the disease progresses, multiple lesions occur, followed by tissue necrosis, reduction in photosynthesis, defoliation, and yield loss (Aristizábal and Johnson, 2022).

A combination of factors, including cultivar susceptibility, plant nutrition, and crop management, contributes to CLR incidence and severity, but environmental variables are also critical (Aristizábal and Johnson, 2022). For example, the urediniospores require temperatures of 17-25°C and leaf wetness for 6–24 hours to germinate (Sera et al., 2022).

While CLR can infect at any time of year, rainfall provides the necessary moisture for urediniospore germination, and the disease usually spreads during the rainy season. Nonetheless, with heavy rain, the urediniospores can be washed away, preventing infection (Sera et al., 2022). In many countries with seasonal rain, the disease reaches its peak intensity in the dry season (Pereira, 1995).

Coffee leaf rust (CLR) is originally from Africa (as is coffee) and was first found in Latin America in 1970 in Bahia, Brazil (Medeiros, 1970). This fungus is highly variable, with more than 50 CLR races identified worldwide, making it increasingly difficult to control with fungicides and necessitating a constant search for new sources of resistance (Carvalho et al., 2011; Talhinhas et al., 2017). Due to the evolution of CLR and dispersal of existing races, resistance in any cultivar and area cannot be assumed to be permanent, and ongoing monitoring is necessary.

Coffee leaf rust was first discovered in Puerto Rico in 1989, and only CLR race II (a race that affects mainly arabica coffee) was identified by CIFC (Centro de Investigação das Ferrugens do Cafeeiro) in 1990 (Macchiavelli and Rodriguez, 2000; Rodríguez et al., 2020). Since then, the fungus rapidly colonized the coffee-growing areas of the west-central mountains, but incidence and severity vary greatly in time and space. Surprisingly, very little has been reported on CLR incidence in Puerto Rico, and nothing is known about CLR severity. The unpredictability of interactions between host genotype, environment, and management means that results from other countries will not necessarily be predictive.

Thirty-six years after its arrival, this is the first study on CLR incidence and severity in Puerto Rico, examining its relationship with environmental conditions. Our study provides the first understanding of CLR disease patterns and variable conditions under which Puerto Rico coffee is grown. Identifying the conditions conducive to CLR can help implement control strategies and reduce losses.

The objective of this study was to assess the temporal dynamics of the CLR and its hyperparasites. The following questions were asked: 1) How do CLR incidence and severity vary between seasons and among years? 2) Are patterns similar between susceptible and resistant coffee cultivars? 3) Which environmental variables are most correlated with CLR incidence and severity? 4) Do the incidence and severity of CLR hyperparasites follow the same patterns as CLR itself?

## 2 Materials and methods

### 2.1 Study area

Sampling to monitor coffee leaf rust (CLR) and its hyperparasites was conducted from March 2022 to February 2025 in fifteen sites. These sites were located on eight private farms and an Agricultural Experiment Station, spanning five municipalities in the coffee-growing area of Puerto Rico: Adjuntas, Ciales, Lares, Orocovis, and Yauco. Sites were chosen to represent a diversity of areas, elevations, cultivars, and management styles; since previous data are extremely limited, the strategy was to cover as wide a range as possible. Sites ranged in elevation from 403 to 951 m above sea level and were planted with eight CLR-susceptible or CLR-resistant cultivars of *Coffea arabica* L. The susceptible cultivars sampled were Caturra, Catuai, Bourbon, Variedad Puerto Rico (VPR) (a type of Typica), and Mundo Novo, while the resistant cultivars were Limaní, Marsellesa, and Obatã (**Table 1**).

**Table 1:** Location, environmental, and management data for the sampled coffee farms.

Municipality	Site	Coordinates N	Coordinates W	Altitude (m a.s.l.)	Coffee cultivar	Age of coffee plants (years)	Management
Adjuntas	S1	18° 10’ 12.46”	66° 47’ 43.38”	633	Catuai	25	Application of Roundup^®^ 4x/year for weed control. Fertilizer NPK 12-5-15, 3x/year For CLR, application of fungicides: Kocide^®^ and Trilogy^®^ once per year.
	S2	18° 10’ 12.41”	66° 47’ 43.42”	590	Caturra	>30	
	S3	18° 10’ 23.71”	66° 47’ 53.48”	577	Limaní	25	
	S4	18° 10’ 37.61”	66° 47’ 25.87”	463	Limaní	>15	Weeds manually removed by trimmer and machete Fertilizer NPK 20-5-20. No CLR management
Ciales	S5	18° 16’ 3.52”	66° 28’ 57.69”	443	Mundo Novo	> 30	Application of Roundup^®^ 4x/year for weed control. Fertilizer NPK 14-7-19. No CLR management
	S6	18° 14’ 53.35”	66° 28’ 33.90”	560	Bourbon	> 25	Application of Roundup^®^ 2x/year for weed control. Fertilizer NPK 20-5-20. No CLR management
Lares	S7	18° 16’ 52.09”	66° 52’ 37.95”	435	Catuai	7	Weeds manually removed by machete Fertilizer NPK 12-5-15, 3x/year For CLR, application of fungicides: Abound^®^ and Cooper Sulfate^®^ 2X per year
	S8	18° 16’ 50.96”	66° 52’ 38.43”	429	Obatã	7	
	S9	18° 16’ 52.29”	66° 52’ 42.26”	419	VPR	> 20	
	S10	18° 16’ 46.96”	66° 52’ 46.49”	403	Marsellesa	4	
Orocovis	S11	18° 13’ 17.42”	66° 29’ 54.40”	458	Catuai, Caturra, Limaní Bourbon	> 10	Weeds manually removed by trimmer and machete Fertilizer NPK 12-5-15. No CLR management
	S12	18° 13’ 22.79”	66° 29’ 58.32”	440	Limaní	6	
Yauco	S13	18° 7’ 58.67”	66° 49’ 23.82”	951	Catuai, Caturra, Limaní	> 30 6	Application of Roundup^®^ 2x/year for weed control. Fertilizer NPK 20-5-20+Mg For CLR, application of fungicides, Kocide^®^ and Kphite^®^ 2x/year.
	S14	18° 7’ 53.01”	66° 50’ 2.77”	783	Caturra, Limaní	uknown	Abandoned farm Application of Roundup^®^ 2x/year for weed control. No CLR management
	S15	18° 8’ 23.41”	66° 50’ 16.12”	889	Caturra, Limaní Bourbon	> 8	Weeds manually removed by trimmer Fertilizer NPK 10-5-15, 3-4x/year No CLR management

Of the fifteen sites, six were in full sun, where the coffee plants grew in monoculture. The remaining nine sites were cultivated under partial shade, primarily provided by *Gliciridia sepium* (madre-de-cacao), *Pithecellobium carbonarium* (carbonero), *Inga vera* (guaba or guama) (all Fabaceae), *Citrus* x *sinensis* (china, Rutaceae), and *Musa acuminata* x *M. balbisiana* (banana or guineo, Musaceae).

At six of the fifteen study sites, fungicides were applied to manage the coffee leaf rust. In Adjuntas (sites S1 and S2), Trilogy^®^ and Kocide^®^; in Lares, (sites S7, S8, and S9), Abound^®^ was applied; and in Yauco, (site S13), Kocide^®^ and Kphite^®^ were applied (**Table 1**).

The environmental variables temperature, relative humidity, dew point, rainfall, and leaf wetness were measured using HOBO RX 2100 weather stations (Onset Computer, Bourne, MA, USA). Temperature, relative humidity, and dew point were also recorded using Onset Hobo MX2301A data loggers. A total of six stations and four data loggers were deployed across ten of the fifteen sites. Environmental variables were not measured at the remaining five sites because they were located on the same farm and were adjacent to other monitored sites. Environmental data were collected from July 2023 to February 2025 and are summarized in **Table 2**, whereas data on CLR incidence, severity, and associated hyperparasites were collected from March 2022 to February 2025.

**Table 2:** Summary of environmental variables by altitude categories for the sites sampled in this study in Puerto Rico.

Altitude	Temperature (°C)			Humidity (%)			Rain (mm)			Leaf wetness (%)	Dew point (°C)
	Min	Mean	Max	Min	Mean	Max	Min	Max	Cumulative	Mean	Mean
Low (403–463 m)	13.2	23.2	33.2	41.6	89.1	100	0	9.0	2,417.4	60.1	20.4
Intermediate (464–633 m)	11.8	22.7	33.2	43.4	94.4	100	0	9.8	2,373.2	78.1	20.7
High (634–951 m)	11.6	21.2	31.8	40.6	89.3	100	0	4.6	2,066.4	64.7	19.1

Means include the entire period from July 2023 to February 2025.

The study area experiences distinct dry and wet seasons, with the dry season occurring from December to March and the wet season from April to November (Jones and Banner, 2003). During this study, the dry season was characterized by lower temperatures, with a minimum of 11.8°C, a mean of 21.4°C, and a maximum of 30.9°C. During this period, precipitation is lower (cumulative 1,845.8 mm), and the frequency of rainfall events was reduced, resulting in decreased relative humidity, which ranged from a minimum of 32.3%, a mean of 72.9%, to a maximum of 100%.

In contrast, the wet season was characterized by higher temperatures, with a minimum of 15.5°C, a mean of 23.7°C, and a maximum of 33.2°C. This period also experienced a substantial increase in precipitation (cumulative 5,608.8 mm) and a corresponding rise in relative humidity, from 56.5% to 100%, with a mean of 85.6%. Notably, cyclonic activity between August and November contributed to the cumulative rainfall of 2,656 mm during these months.

### 2.2 CLR Incidence, severity, and plant defoliation

CLR damage was surveyed every three weeks at each site, for a total of 46 sampling dates per site over three years. CLR damage was assessed using two standard epidemiological variables: incidence and severity (Hughes, 1999). Incidence was defined as the proportion of plant units (e.g., leaves or branches) with visible CLR lesions, relative to the total number of units assessed. Severity was defined as the proportion of leaf area with CLR lesions, relative to the total leaf area.

To determine the incidence of CLR and its hyperparasites, twenty-five coffee plants were selected at each site. For each plant, one branch was chosen at the lower (<1m), middle (1-2m), and upper (>2m) canopy levels. For each branch, the number of fully developed leaves and immature leaves, leaves showing CLR symptoms (with sporulated lesions), and the number of CLR-infected leaves with hyperparasites were counted. The incidence of CLR and its hyperparasites was calculated using the following equations:


$$\text{CLR-incidence}=\left(\;\frac{\text{Number of coffee leaves infected with the CLR}}{\text{Total number of fully developed leaves per branch }}\right)*100$$

$$\text{Incidence of hyperparasites}=\left(\;\frac{\text{Number of coffee leaves infected with CLR, with white mycelium growing on CLR lesions}}{\text{Number of coffee leaves infected with the CLR }}\right)*100$$

Severity was determined by randomly collecting nine infected leaves from each canopy level (lower, middle, and upper), for a total of twenty-seven leaves per site per sample date. In the lab, the abaxial surface of each leaf was photographed. Using the software Image J (National Institutes of Health, USA), the total leaf area, the area infected with the CLR, and the CLR-infected area colonized by hyperparasites were measured using the threshold function (Aristizábal and Johnson, 2022). Severity of CLR and its hyperparasites was calculated using the following equations:


$$\text{CLR severity}=\left(\;\frac{\text{Coffee leaf area infected with CLR}}{\text{Total leaf area }}\right)*100$$

$$\begin{array}{c} \text{Severity of hyperparasites}=\\ \left(\;\frac{\text{Area of white mycelium growing on CLR-infected areas}}{\text{Coffee leaf area infected with CLR}}\right)*100 \end{array}$$

In the field, hyperparasites were visually identified as white mycelium growing on CLR lesions (**Figure 1**). In the laboratory, conidia of hyperparasites were collected from a selection of parasitized pustules using a sterilized glass Pasteur pipette with the tip drawn to a fine point. Conidia were plated onto potato dextrose agar (PDA) amended with 0.125 g/L streptomycin and 0.025 g/L ampicillin. Cultures were incubated at room temperature (23 ± 2°C) for two weeks. Isolates exhibiting characteristics of Cordycipitaceae (white, abundant, fluffy, and compact aerial mycelium with slow growth) were subcultured to obtain pure cultures. Under the microscope, the shape of conidia, conidiophores, and with conidiogenous cells was examined. *Lecanicillium-*like hyperparasites had short-ellipsoidal to subcylindrical conidia and erect conidiophores with one or two whorls of phialides (Zare and Gams, 2001).

Plant defoliation was visually estimated as the percent leaf loss per plant, ranging from 0% (no defoliation) to 100% (complete defoliation), in increments of 5%.

### 2.3 Statistical analysis

A descriptive analysis with the estimated monthly means of CLR incidence and severity was conducted to identify temporal changes in CLR over the three-year sampling period. Elevation was divided into three classes using the Jenks natural breaks classification (Jenks, 1967), which minimizes within-group variance and maximizes between-group variance. Based on this classification, sites were categorized as low (403–463 m a.s.l.), intermediate (464–633 m a.s.l.), and high elevation (634–951 m).

To determine the effect of coffee farm management practices on CLR incidence and severity, data from the fifteen sites were divided into five groups according to the practices employed by each grower: 1) where fungicides, fertilizers, and herbicides were applied; 2) where fungicides and herbicides were applied; 3) where fertilizers and herbicides were applied; 4) where only fertilizers were applied; 5) an abandoned site, where herbicides were applied only occasionally for weed control (**Table 1**).

To determine the effect of fungicide applications on CLR incidence and severity, data from sites S1, S2, S7, S8, and S9 were used; data from S13 were excluded because the grower did not record the dates of fungicide application. For each site, CLR incidence and severity data from three sampling dates before fungicide application were compared with data from three sampling dates after the application.

The response variables were incidence and severity of the CLR and its hyperparasites. The explanatory variables were years (only 2022, 2023, and 2024 were included, as in 2025, only January and February were sampled), branch position (lower, middle, and upper), seasons (wet vs dry), age of coffee plants, management practices, fungicide application and coffee cultivar (susceptible vs. resistant), as classified by World Coffee Research (WCR, 2025). Sampling time was treated as a repeated measure for each branch and leaf, accounting for the hierarchical structure of the data.

Data was analyzed using generalized linear mixed models (GLMM). For incidence data, models were fitted with a binomial error distribution and a logit link function, which is appropriate for binary variables (e.g., number of infected vs. uninfected leaves). For severity data, models were fitted with a beta error distribution and a logit link function, suitable for continuous proportional data bounded between 0 and 1.

For all models, the response variable (Y*_ij_*) was defined as the number of leaves or the proportion of leaf area infected by the CLR, or as the number of CLR- infected leaves or proportion of CLR- infected area hyperparasitized, in a branch or leaf *j* of a plant *i*, conditional on the total number of leaves or leaf area observed (*n_ij_*), and on the probability that a coffee leaf is infected by the CLR or that a CLR lesion is hyperparasitized (*p_i_*). Specifically:

CLR incidence: Y*_ij_* = number of CLR-infected leaves in a branch *j* of the plant *i*, *n_ij_* = total number of leaves in a branch *j*, *p_i_*= probability that a coffee leaf is infected with CLR.
Incidence of hyperparasites: Y*_ij_*_=_ number of coffee leaves with hyperparasitized CLR lesions in a branch branch *j* of the plant *i*, *n_ij_* = total number of CLR-infected leaves in a branch *j*, *p_i_*= probability that a CLR lesion is hyperparasitized in a branch.
CLR severity: Y*_ij_* = proportion of leaf area infected by the CLR on leaf *j* of plant *i*, *n_ij_* = total area of the coffee leaf *j*, *p_i_*= probability that a unit of leaf area is infected with CLR.
Severity of hyperparasites: Y*_ij_* = proportion of CLR-infected area hyperparasitized on leaf *j* of plant *i*, *n_ij_* = total CLR-infected area on leaf *j*, *p_i_*= probability that a unit CLR-infected area is hyperparasitized.

The GLMM for all four variables (CLR incidence, incidence of hyperparasites, CLR severity and severity of hyperparasites) can be expressed in the following equation:


$$Y_{ij}∼\text{Binomial}\;({\text{n}}_{\text{i}\text{j}},{\text{p}}_{\text{i}})$$

with the linear predictor:


$$\;\;\mathrm{logit}\;\left({\text{p}}_{\text{i}}\right)=\;{\text{β}}_{0}\;+{\text{β}}_{1}\;+\;\underset{k}{\sum }\mu k_{\left[\text{i}\right]}$$

where: $\beta_{0}$ is the model intercept, $\beta_{1}$ represents the fixed effect of explanatory variables (e.g., resistant vs. susceptible cultivars, years, seasons, coffee cultivars, branch position in a coffee plant).

µ*_k_*_[i]_ denotes the random intercept associated with the *k*^th^ grouping factor for observation *i*.

The summation term accounts for random variation, which may arise from factors such as year, season, coffee cultivar, management practices, altitude, and plant age, depending on the specific model and explanatory variables included in each one.

GLMM models were fitted using the glmmTMB package in R (Bolker, 2023; Brooks et al., 2025). This tool provides robust handling of zero-inflated datasets, particularly those involving disease count data. Tukey’s *post hoc* tests were performed at a significance level of 0.05 to assess differences among resistant vs susceptible cultivars, branch positions in a plant, altitude, seasons, and coffee cultivars. These comparisons were conducted using the glht() function from the multcomp package (Hothorn et al., 2008).

Pearson’s correlation analyses were performed to determine the relationships among CLR and hyperparasite variables: incidence and severity, with environmental variables: minimum, maximum, mean, and median temperature, and mean relative humidity, dewpoint, rainfall, and leaf wetness, based on triweekly averages of measurements of each environmental variable before the sampling. Also, Pearson’s correlations were done to determine the relationships between: CLR incidence and CLR severity, CLR incidence and severity and plant defoliation, CLR incidence and incidence of hyperparasites, and CLR severity and severity of hyperparasites. For correlation analyses, data on CLR and hyperparasite incidence and severity were square root transformed. All analyses were performed using R version 4.3.0 (R Core Team, 2023).

All data are presented as mean ± standard error.

## 3 Results

### 3.1 Seasonal variation in CLR

**Figure 2:**
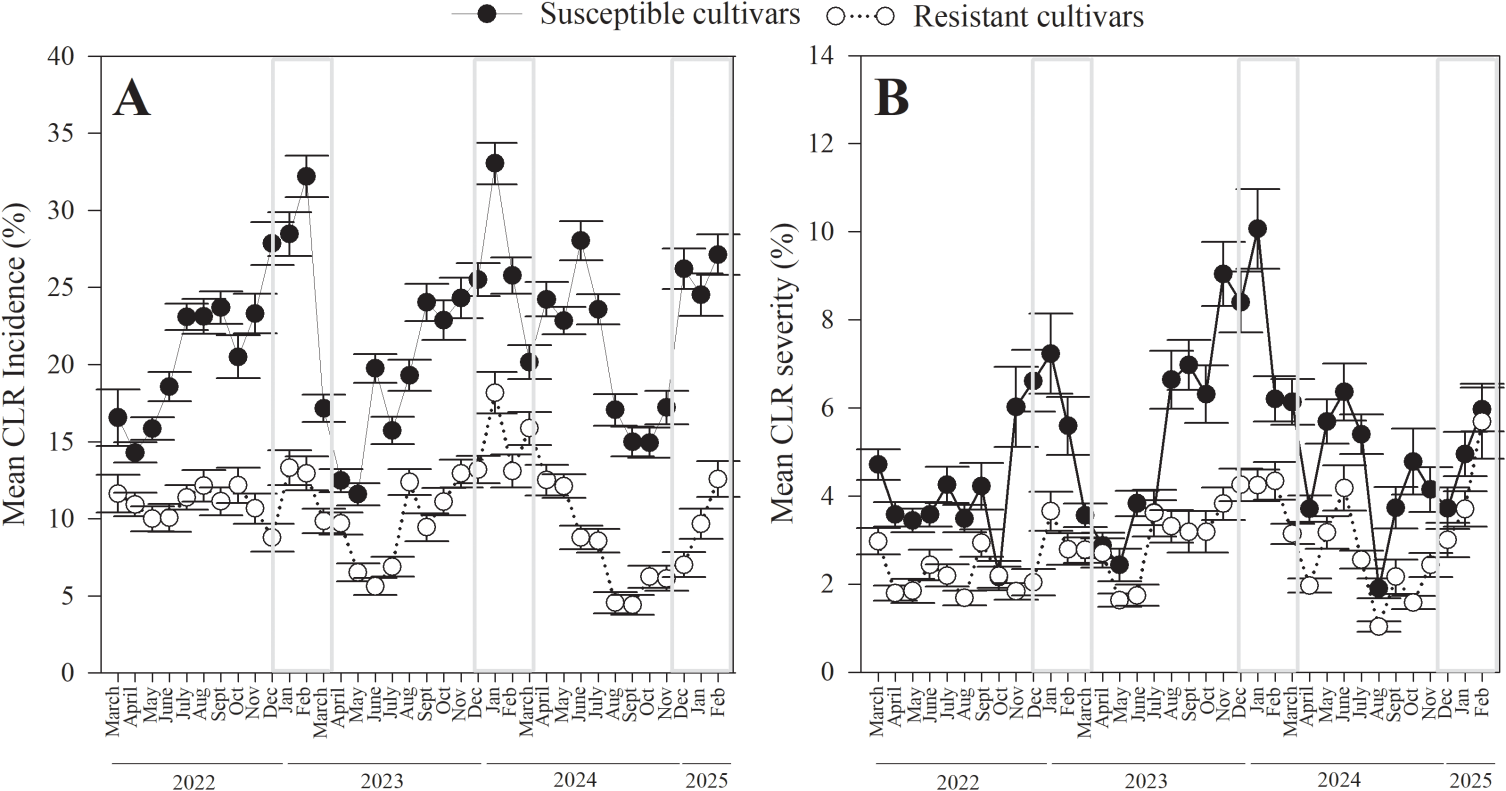
Temporal dynamics of coffee leaf rust (*H. vastatrix*) incidence and severity over three years of sampling across fifteen coffee sites in Puerto Rico, comparing susceptible and resistant coffee cultivars. **(A)** CLR incidence, or percent coffee leaves with CLR lesions per branch, **(B)** CLR severity, or percent coffee leaf area with CLR lesions. Gray boxes show the dry seasons.

A temporal pattern was observed in both CLR incidence and severity. Overall, CLR was lowest from April to May (**Figures 2A, B**). During these months, the mean incidence was < 24.2 ± 0.35% for susceptible, and < 12.5 ± 0.21% for resistant cultivars. Similarly, severity was < 5.7 ± 0.12% for susceptible, and < 3.1 ± 0.06% for resistant cultivars. A significant increase in both CLR incidence and severity was observed starting in June, with a marked peak during January and February. The mean incidence reached 33.1 ± 0.53% for susceptible cultivars during February, and 6.8 ± 0.38% in January for resistant cultivars. Severity peaked in January, reaching 10.1 ± 0.28% for susceptible and 5.7 ± 0.12% for resistant cultivars. This seasonal pattern was consistent across all three years.

Comparisons of CLR incidence and severity across years, including data from both susceptible and resistant cultivars, showed significantly higher values in 2024 than in 2023 and 2022 (incidence: χ^2^_2_ = 236.4, *p* < 0.001; severity: χ^2^_2_ = 19.1, *p* < 0.001). CLR incidence across the three full years ranged from 0 to 100%, mean incidence was 16.2 ± 0.21% in 2022, 20.7 ± 0.26% in 2023, and 22.5 ± 0.30% in 2024. Mean severity was 3.5 ± 0.07% in 2022, 4.6 ± 0.08% in 2023, and 4.9 ± 0.09% in 2024.

These differences among years remained significant when data from susceptible and resistant cultivars were analyzed separately (incidence: susceptible, χ^2^_2_ = 263.2, *p* < 0.001; resistant, χ^2^_2_ = 117.6, *p* < 0.001); (severity: susceptible, χ^2^_2_ = 27.8, *p* < 0.001; resistant, χ^2^_2_ = 14.2, *p* < 0.001) (**Figure 2**).

#### 3.1.1 Dry vs. wet season

**Figure 3:**
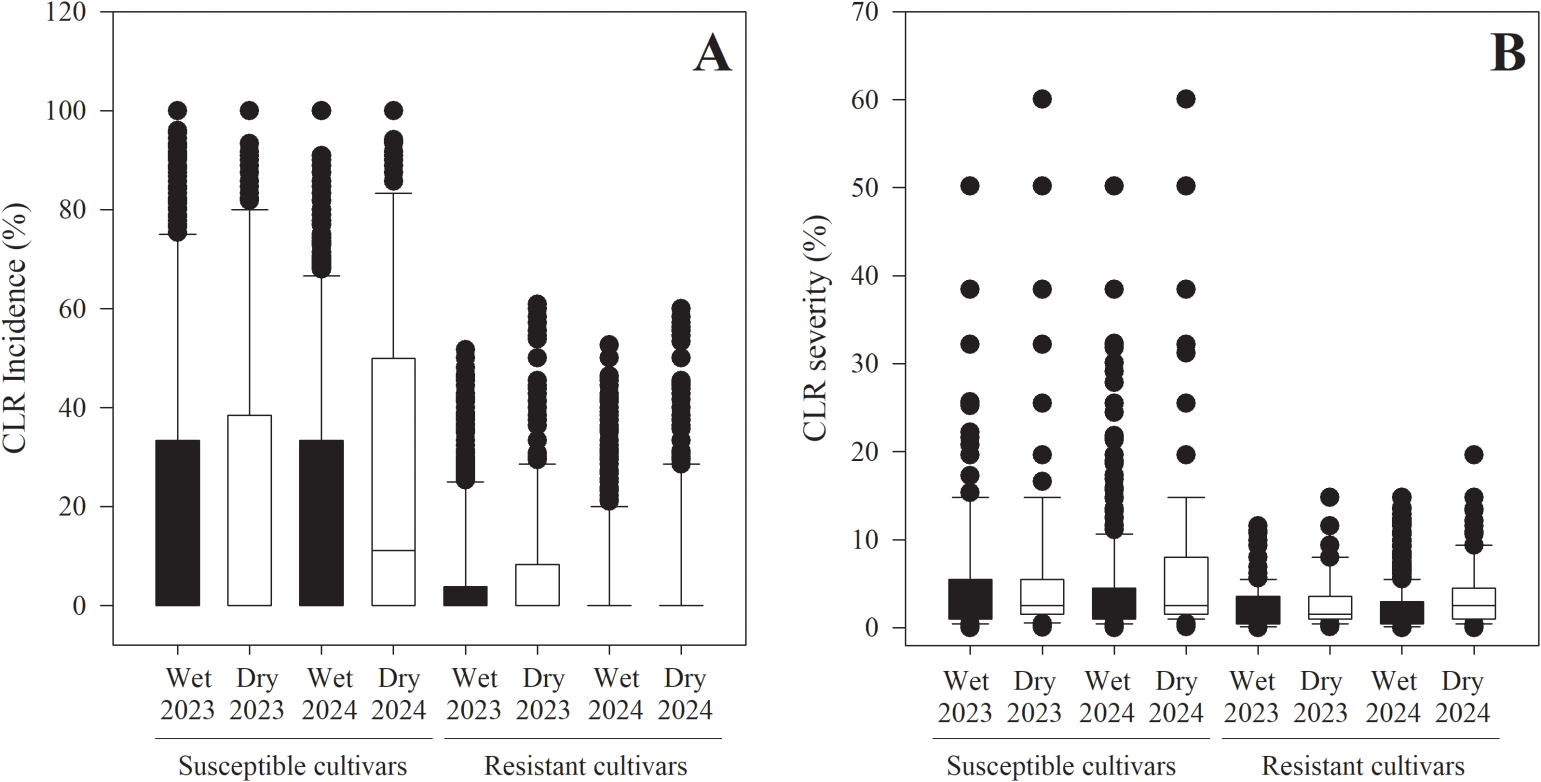
Differences in coffee leaf rust (*H. vastatrix*) incidence and severity between wet and dry seasons and between susceptible and resistant coffee cultivars. The wet season is April to November, while the dry season is December to March **(A)** Boxplots of CLR incidence (% infected leaves per branch), **(B)** Boxplots of CLR severity (% leaf area infected). In each boxplot, the horizontal line inside the box represents the median; the bottom and top lines indicate the 25th (Q1) and 75th (Q3) percentiles, respectively. The whiskers represent minimum and maximum values within 1.5x the interquartile range, and the black dots indicate outliers beyond this range.

CLR incidence and severity were significantly higher in the dry season than in the wet season for both susceptible and resistant cultivars, incidence (susceptible: χ^2^_1_ = 330.5, *p<* 0.001; resistant: χ^2^_1_ = 231.4, *p* < 0.001), and severity (susceptible: χ^2^_1_ = 34.8, *p* < 0.001; resistant: χ^2^_1_ = 44.7, *p* < 0.001) (**Figures 3A, B**). CLR incidence for susceptible cultivars ranged from 0 to 100% across both seasons, with a mean incidence of 26.2 ± 0.42% in the dry season and 20.7 ± 0.27% in the wet season. In resistant cultivars, incidence ranged from 0 to 61.5% during the dry season and from 0 to 52.6% during the wet season, with mean values of 6.8 ± 0.21% and 5.4± 0.12% for the dry and wet seasons, respectively.

CLR severity in susceptible cultivars ranged from 0 to 60.1% during the dry season, and from 0 to 50.1% during the wet season, with a mean severity of 6.4 ± 0.16% and 5.4 ± 0.04%, respectively. In resistant cultivars, severity ranged from 0 to 19.6% during the dry season and from 0 to 14.8% during the wet season, with a mean of 3.3 ± 0.08% in the dry season and 2.2 ± 0.04% in the wet season.

### 3.2 Branch position within the coffee plant

CLR incidence and severity were higher on lower and middle branches than on upper branches (incidence: χ^2^_2 =_ 1,184.3, *p<* 0.001; severity: χ^2^_2 =_ 19.7, *p<* 0.001). CLR incidence ranged from 0 to 100% for branches located at the three positions in a coffee plant. However, branches located at upper canopy positions had 63% of the branches evaluated with a CLR incidence of less than 5%. Mean incidence was 16.5 ± 0.21% for lower branches, 16.2 ± 0.21% for middle branches, and 12.7 ± 0.19% for upper branches. CLR severity ranged from 0 to 63.9% for branches at the lower canopy level, 0 to 60.1% for branches at the middle canopy level, and 0 to 35.9% for branches at the upper canopy level, with mean values of 4.5 ± 0.08%, 4.1 ± 0.09%, and 3.8 ± 0.07%, respectively. All other analyses presented here combine data for all three levels.

### 3.3 Altitude

**Figure 4:**
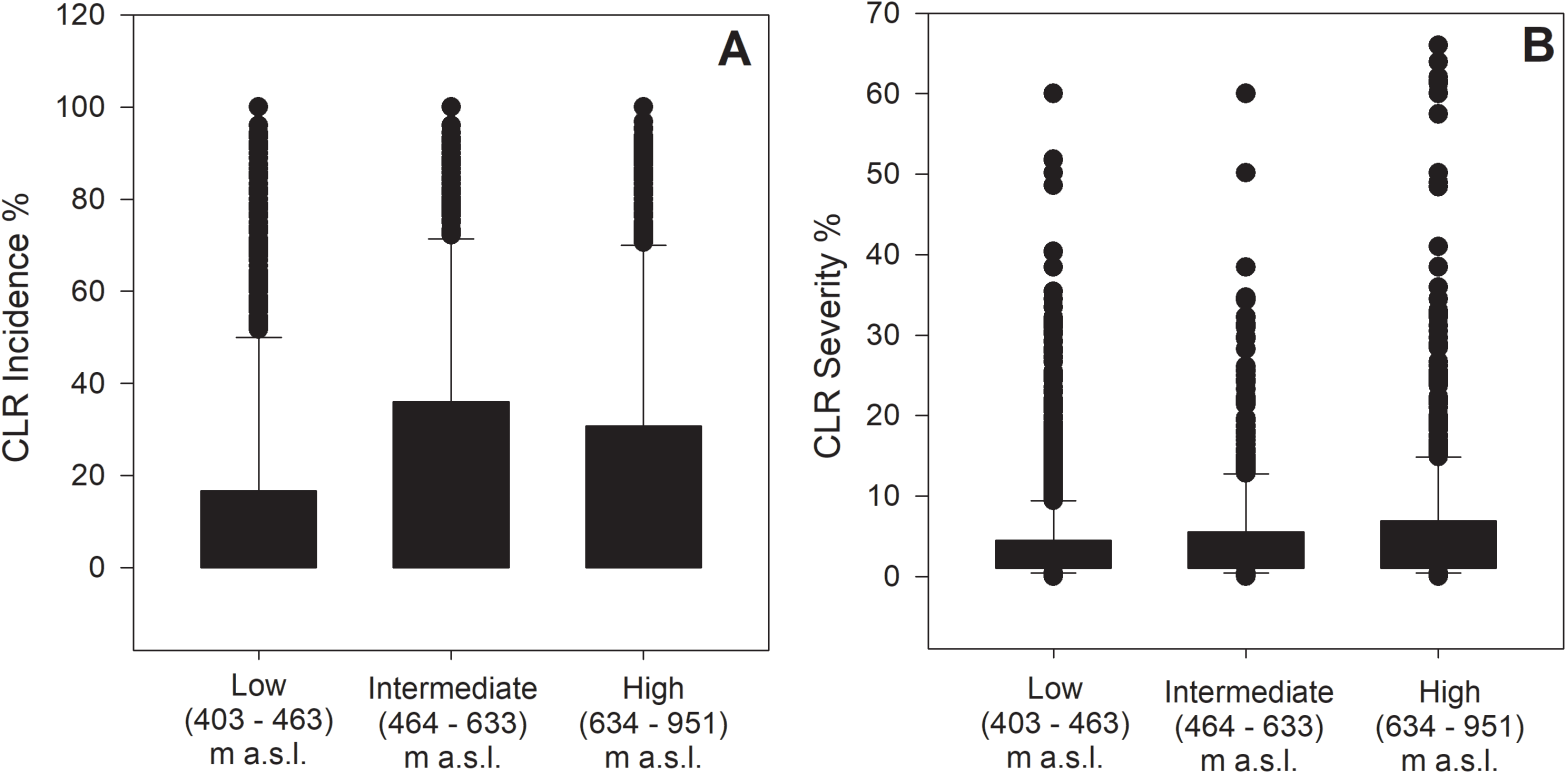
Differences in coffee leaf rust (*H. vastatrix*) incidence and severity among categories of altitude. **(A)** Boxplots of CLR incidence (% infected leaves per branch), **(B)** Boxplots of CLR severity (% leaf area infected). In each boxplot, the horizontal line inside the box represents the median; bottom and top lines indicate the 25th (Q1) and 75th (Q3) percentiles, respectively. The whiskers represent minimum and maximum values within 1.5x the interquartile range and the black dots indicate outliers beyond this range.

CLR incidence and severity increased significantly with altitude (incidence: χ^2^_2_ = 5.6, *p* = 0.01; severity: χ^2^_2_ = 4.4, *p* = 0.01); CLR incidence ranged from 0 to 100% for the three categories of altitude. However, 81.5% of branches evaluated at sites located ≤ 463 m a.s.l. (‘low’) had < 25% of incidence, the mean was 11.5 ± 0.13% for sites located at (‘low’) altitudes, 20.9 ± 0.26% for sites located at 464 m a.s.l. (‘intermediate’) and 20.2 ± 0.31% at ≥ 634 m a.s.l. (‘high’). Differences in incidence between intermediate and high altitudes were not significant (*p* = 0.106) (**Figure 4A**). CLR severity ranged from 0 to 50.2% at sites at lower altitudes, with a mean severity of 3.5 ± 0.05%, at intermediate and high altitudes, severity ranged from 0 to 60.1%, with mean severity values of 4.6 ± 0.10% and 5.8 ± 0.16%, respectively (**Figure 4B**); as observed for incidence, differences between intermediate and high altitudes were not significant (*p* = 0.323).

### 3.4 Differences among *Coffea arabica* cultivars

**Figure 5:**
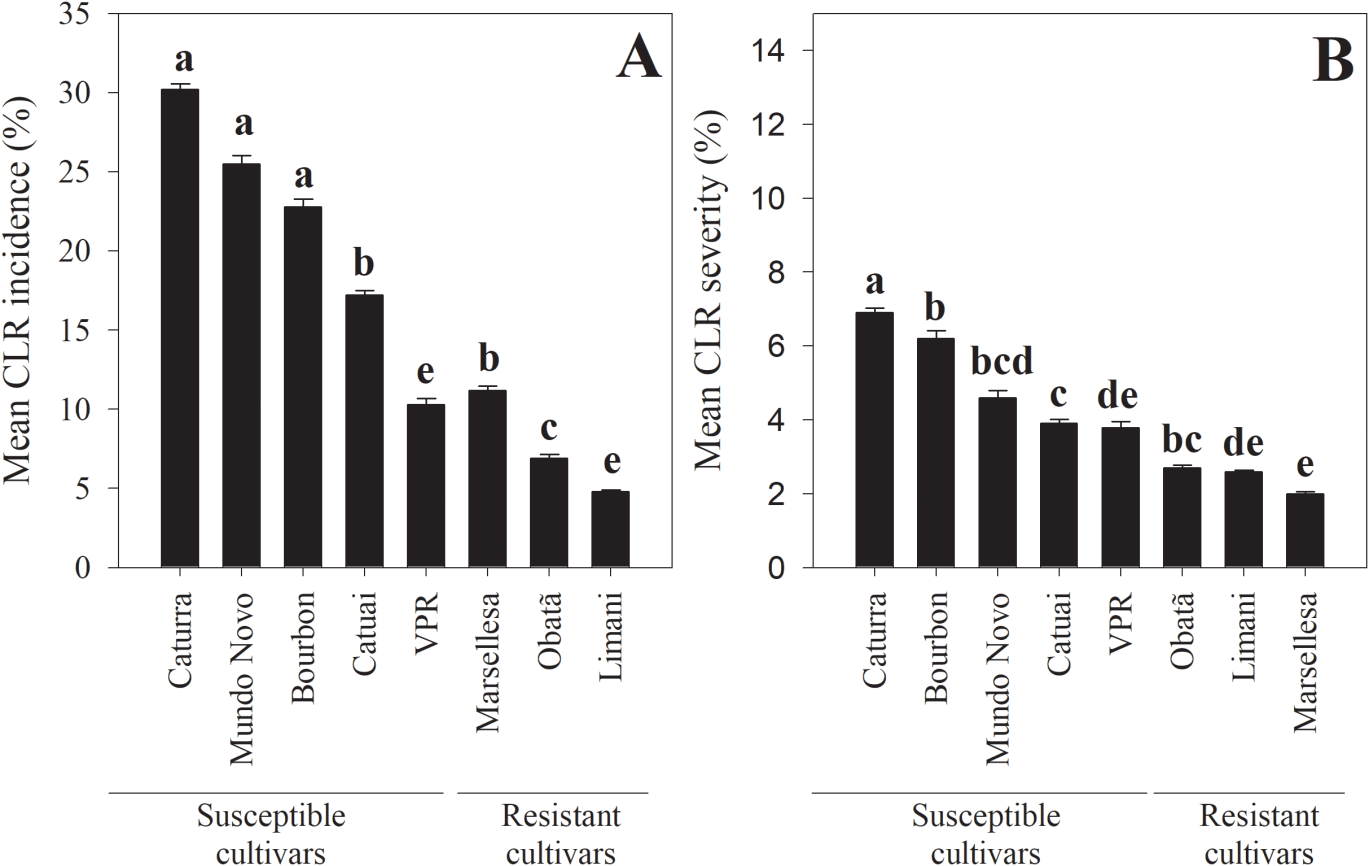
Differences in coffee leaf rust (*H. vastatrix*) incidence and severity comparing among susceptible and resistant coffee cultivars. **(A)** CLR incidence, or percent coffee leaves with CLR lesions per branch, **(B)** CLR severity, or percent coffee leaf area with CLR lesions. Bars with different letters are statistically different based on Tukey's HSD post hoc tests (p<0.05).

CLR Incidence and severity were two-fold higher in susceptible cultivars than in resistant ones. Both differences were significant (incidence: χ^2^_2_ = 615.2, *p* < 0.001; severity: χ^2^_2_ = 146.7, *p* < 0.001). CLR incidence in susceptible cultivars ranged from 0 to 100%, 32% of the evaluated branches showed incidence levels > 25%, with a mean of 22.1 ± 0.18%. In resistant cultivars, incidence ranged from 0 to 61.5%, with only 13% of branches with incidence > 25%, and a mean of 6.1 ± 0.09%. (**Figure 5A**).

CLR severity in susceptible cultivars ranged from 0 to 60.1%, with a mean of 5.3 ± 0.07%; 46% of the evaluated leaves showed severity > 2.5%. In contrast, in resistant cultivars, severity ranged from 0 to 19.6%, with a mean of 2.5 ± 0.03%. Only the 29% of leaves had severity > 2.5%.

Among the susceptible cultivars, Caturra was highest in CLR incidence and severity, followed by Bourbon, Mundo Novo, and Catuai, whereas VPR had lower incidence and severity (incidence: χ^2^_4_ = 731.9, *p* < 0.001; severity: χ^2^_4_ = 23.1, *p* = 0.004). CLR incidence among the five susceptible cultivars ranged from 0 to 100%, with a mean incidence in Caturra of 30.2 ± 0.38%, in Mundo Novo 25.5 ± 0.54%, in Bourbon 22.7 ± 0.51%, in Catuai 17.2 ± 0.28%, and in VPR 10.3 ± 0.37% (**Figure 5A**).

Severity ranged from 0 to 60.1%. The highest severity was observed in leaves of Caturra, followed by Bourbon. In both cultivars, severity reached a maximum of 60.1%, with mean of 6.9 ± 0.13% and 6.2 ± 0.21%, respectively. In the other three cultivars, maximum severity reached 50.2%, with means in Mundo Novo of 4.6 ± 0.19%, in Catuai of 3.9 ± 0.11%, and in VPR of 3.7 ± 0.17% (**Figure 5B**).

Among the resistant cultivars, Marsellesa and Obatã had higher CLR incidence than Limaní (χ^2^_2_ = 15.8, *p* < 0.001). CLR incidence in the three resistant cultivars ranged from 0 to 60%, with a mean in Marsellesa of 11.2 ± 0.28%, followed by Obatã with 6.9 ± 0.24%, and Limani with 4.8 ± 0.10% (**Figure 5A**).

CLR severity was significantly higher in Obatã than in Marsellesa and Limani (χ^2^_2_ = 28.9, *p* < 0.001). Severity in Obatã ranged from 0 to 14.1%, with a mean of 2.7 ± 0.08%, followed by Limani, which ranged from 0 to 19.6%, with a mean of 2.6 ± 0.04%. In Marsellesa, severity ranged from 0.04 to 14.8%, with a mean of 2.0 ± 0.06% (**Figure 5B**). Severity values were low (< 2.5%) in more than 67% of leaves evaluated in Obatã, 71% in Limani, and 80% in Marsellesa.

### 3.5 Coffee farm management and fungicide applications

Fungicide applications were associated with a significant reduction in CLR incidence and severity, both by approximately 50% (incidence: χ^2^_1_ = 157.8, *p* < 0.001; severity: χ^2^_2_ = 29.4, *p* < 0.001). Mean CLR incidence was 10.1 ± 0.50% before fungicide application and 4.9 ± 0.29% after application. Mean CLR severity decreased from 4.1 ± 0.26% before application to 2.9 ± 0.20% after application.

Contrary to fungicide applications, management practices did not appear to be related to CLR incidence and severity (incidence: χ^2^_4_ = 4.2, *p* = 0.386; severity: χ^2^_4_ = 4.1, *p* = 0.388). Mean CLR incidence and severity were 21.3 ± 0.26% and 4.4 ± 0.10%, respectively at sites where fungicides, fertilizers, and herbicides were applied; 13.7 ± 0.17% and 3.1 ± 0.06% at sites where fungicides and fertilizer were applied, 24.2 ± 0.38% and 5.2 ± 0.14% at sites where fertilizers and herbicides were applied, 8.8 ± 0.16% and 4.1 ± 0.07% at sites where only fertilizers were applied; and 22.9 ± 0.62% and 6.3 ± 0.29%, at the abandoned site, where herbicides were applied only occasionally for weed control.

### 3.6 Coffee plant age

The effect of coffee plant age on CLR was not clear. No significant differences were observed in CLR incidence (χ^2^_8_ = 10.1, *p* = 0.349). Although significant differences were observed in severity (χ^2^_8_ = 114.1, *p* < 0.001), there was no consistent pattern of CLR damage increasing with plant age. Mean incidence was 13.7 ± 0.28%, 5.1 ± 0.15%, 13.1 ± 0.24%, 13.4 ± 0.41%, 11.2 ± 0.40%, 10.3 ± 0.26%, 10.4 ± 0.0.37%, 16.4 ± 0.27%, and 29.9 ± 0.36% in four-, six-, seven-, eight-, ten-, fifteen-, twenty-one-, twenty-five-, and thirty-year-old plants, respectively. Mean severity was 2.0 ± 0.06%, 2.5% ± 0.08, 3.4 ± 0.09%, 6.3% ± 0.29, 5.9% ± 0.20, 3.1 ± 0.07%, 3.8 ± 0.17%, 3.9 ± 0.10% and 6.3% ± 0.14 in plants of the same age classes, respectively.

### 3.7 Coffee plant defoliation

**Figure 6:**
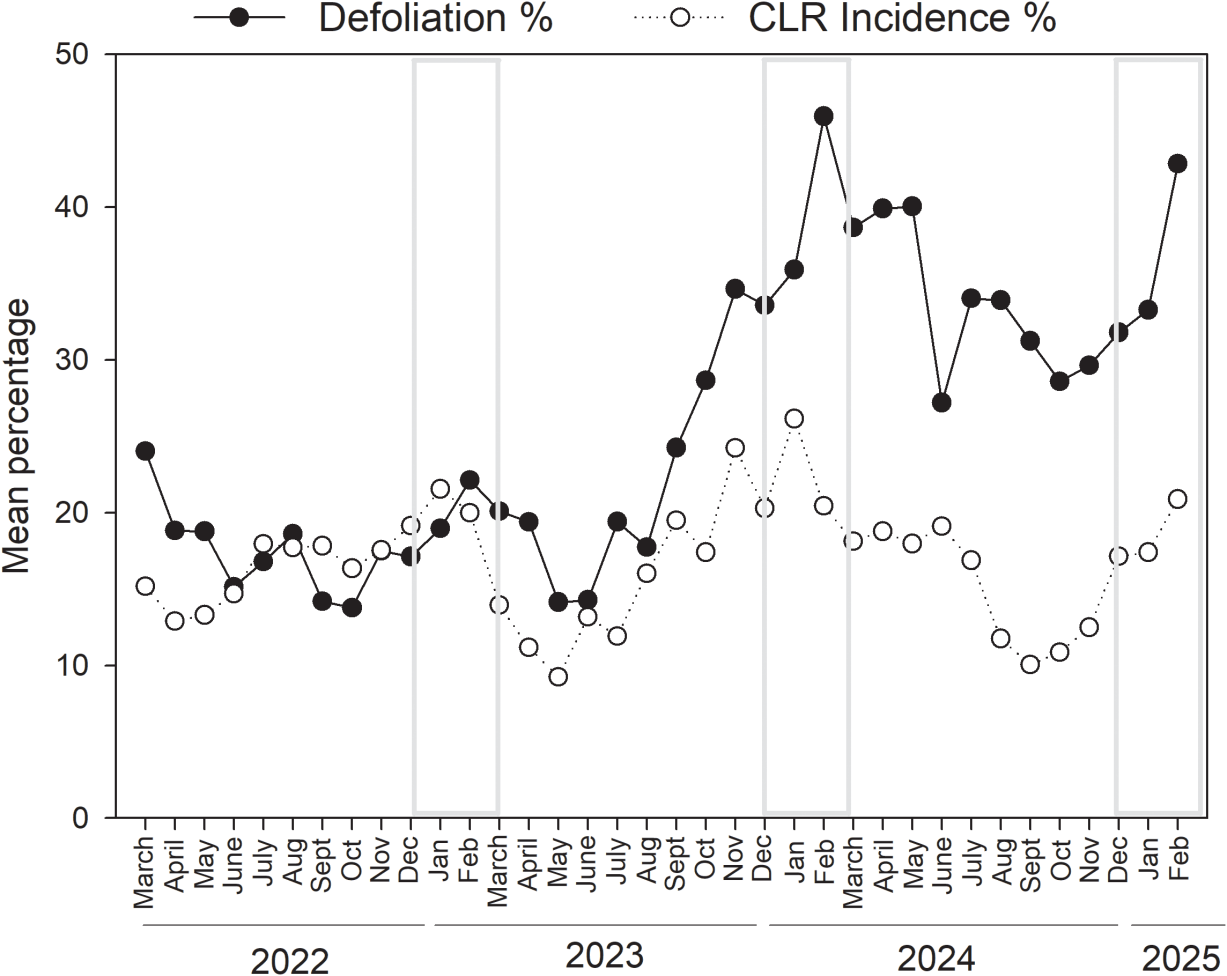
Temporal dynamics of plant defoliation and coffee leaf rust (*H. vastatrix*) incidence over three years of sampling across fifteen coffee sites in Puerto Rico. Gray boxes show the dry seasons.

Defoliation over time closely mirrored the temporal patterns observed for CLR incidence (**Figure 6**), ranging from 0 to 98%. Defoliation increased over the years, with the highest levels observed in 2024, followed by 2023 and 2022. The mean defoliation for each year was 37.4 ± 0.48, 24.1 ± 0.39, and 18.8± 0.37%, respectively. Overall, the lowest levels of defoliation were observed from April to June (**Figure 6**). During these months in 2022 and 2023, the mean defoliation was 15.8 ± 0.70 -20.1 ± 0.74%, while in 2024 it was 32.8 ± 1.44% - 41.7 ± 1.70%. A significant increase in defoliation was observed from September to October, peaking in February with the highest mean values of 45.9 ± 1.77% in 2024 and 42.8 ± 1.92% in 2025. This seasonal pattern was consistent across all three years. Defoliation was significantly higher during the dry season than the wet season (χ^2^_1_ = 5.7, *p* = 0.01). Mean defoliation was 33.4 ± 0.49% for the dry season and 25.6 ± 0.29% for the wet season.

### 3.8 Hyperparasites

**Figure 7:**
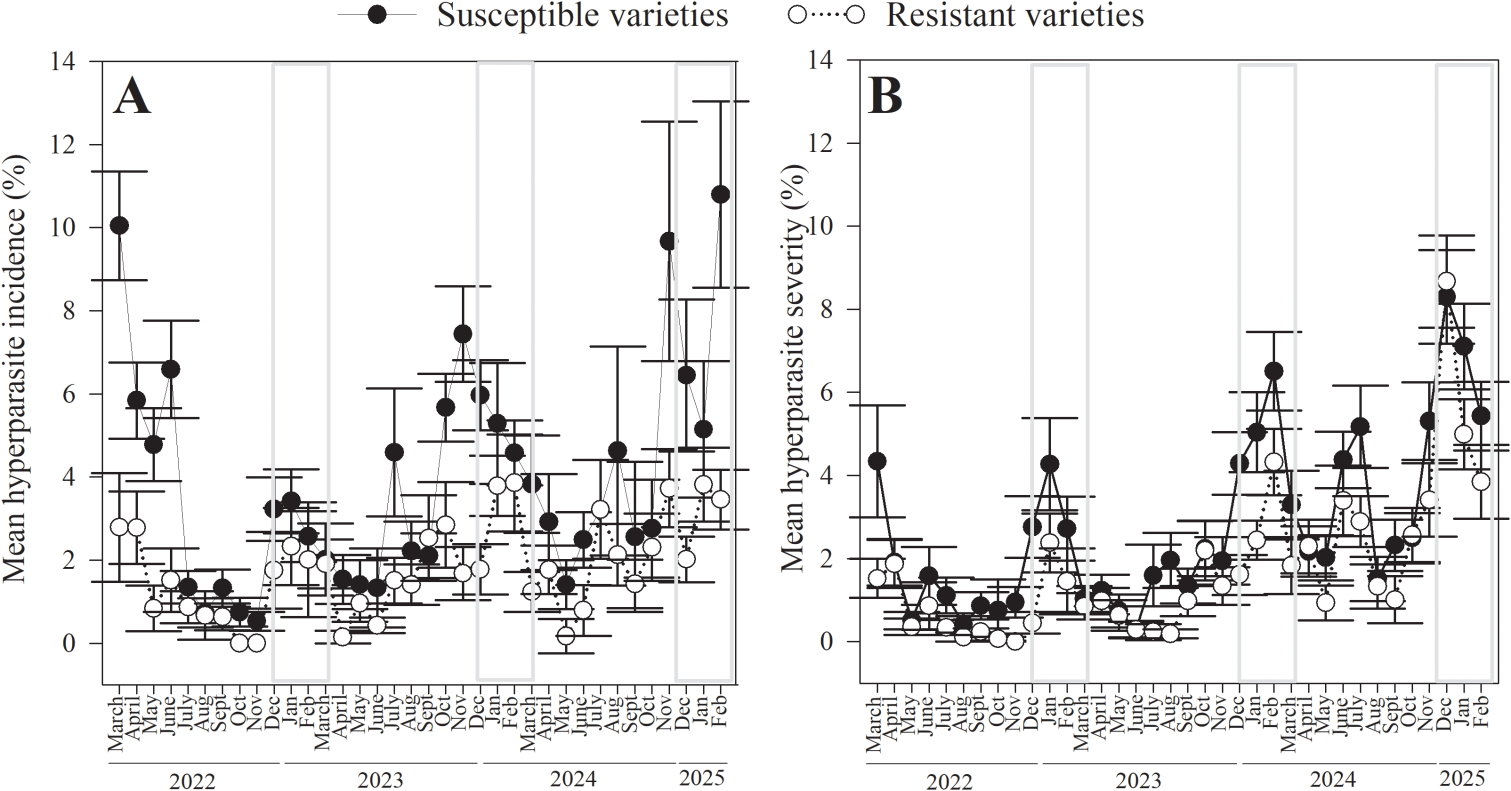
Temporal dynamics of hyperparasites of coffee leaf rust (*H. vastatrix*): incidence and severity over three years of sampling across fifteen coffee sites in Puerto Rico, comparing susceptible and resistant coffee cultivars. **(A)** Hyperparasite incidence, or percent coffee leaves with CLR lesions that had visible hyperparasite colonies. **(B)** Hyperparasite severity, or percent area of CLR lesions hyperparasitized. Gray boxes show the dry seasons.

Changes in incidence and severity of hyperparasites over time closely mirrored the temporal patterns observed for CLR (**Figures 7A, B**). Overall, both incidence and severity of hyperparasites were lowest from May to September, followed by a notable increase beginning in October. Two distinct peaks in hyperparasites activity were observed, the first from November to December, and the second from February to March. The highest incidence of hyperparasitism was recorded for susceptible cultivars in March 2022 (10.5 ± 1.07%), November 2024 (9.7 ± 0.98%), and February 2025 (10.8 ± 0.72%). For severity, peak values were observed in February 2024 (6.3 ± 0.64%), December 2024 (8.3 ± 0.79%), and January 2025 (7.1 ± 0.67%).

Both incidence and severity of hyperparasites were significantly higher in susceptible than in resistant cultivars (incidence: χ^2^_1_ = 64.1, *p* < 0.001; severity: (χ^2^_1_ = 1.48, *p* = 0.026). Incidence of hyperparasites in both types of cultivars ranged from 0 to 100%, with a mean of 3.0 ± 0.12% in susceptible cultivars and 2.3 ± 0.16% in resistant cultivars. Only 6.0% and 4.7% of the evaluated branches in susceptible and resistant cultivars, respectively, had leaves with CLR lesions colonized by hyperparasites.

Severity of hyperparasites in susceptible cultivars ranged from 0 to 100%, with a mean of 6.1 ± 0.11%, while severity in resistant cultivars varied from 0 to 60.1%, with a mean of 3.1 ± 0.11%. Similar to the incidence of hyperparasites, their severity was also low: only 10% and 8.1% of the evaluated leaves in susceptible and resistant cultivars, respectively, had CLR lesions colonized by hyperparasites.

Like CLR, incidende and severity of hyperparasites were significantly higher during the dry season than in the wet season (incidence: χ^2^_1 =_ 6.0, *p* = 0.01; severity: χ^2^_1 =_ 6.2, *p* = 0.01). Incidence of hyperparasites in both seasons ranged from 0 to 95%, with a mean of 3.7 ± 0.20% in the dry season and 2.9 ± 0.11% in the wet season. Severity of hyperparasites ranged from 0 to 90%, with a mean of 3.9 ± 0.17% in the dry season and 1.6 ± 0.08% for the wet season.

Regarding canopy level, both incidence and severity of hyperparasites were significantly higher on branches and leaves positioned on the lower levels than on middle and upper (incidence: χ^2^_2 =_ 415.5, *p<* 0.001; severity: χ^2^_2 =_ 23.7, *p<* 0.001). Incidence of hyperparasites ranged from 0 to 100% across the three canopy levels, with mean values of 3.5 ± 0.19%, 2.9 ± 0.16%, and 1.9 ± 0.15% in lower, middle, and upper branches, respectively. Hyperparasites were observed on 6.6% of lower, 6.1% of middle, and 3.9% of upper branches. Severity of hyperparasites ranged from 0 to 100% on leaves at the lower canopy level, 0 to 95% at the middle level, and 0 to 90% at the upper level, with means of 3.2 ± 0.16%, 2.3 ± 0.14%, and 1.6 ± 0.11%, respectively. Consistent with incidence patterns, hyperparasites were more frequently observed on leaves at the lower canopy levels (10.8%), followed by middle (9.3%), and upper levels (7.1%).

### 3.9 Correlations between CLR, hyperparasites and environmental variables

Correlation analyses showed significant positive associations between CLR and several biotic and environmental variables. CLR incidence was positively correlated with CLR severity (r = 0.48, p < 0.001), plant defoliation (r = 0.38, p < 0.001), and incidence of hyperparasites (r = 0.24, p <0.001). Similarly, CLR severity was positively correlated with plant defoliation (r = 0.21, p < 0.001) and severity of hyperparasites (r = 0.16, p <0.001).

#### 3.9.1 CLR and temperature

Significant negative correlations were found between CLR incidence and various temperature metrics, mean (r = -0.37, p<0.001), median (r = -0.40, p<0.001), minimum (r = -0.20, p=0.004), and maximum (r = -0.29, p< 0.001). Similarly, CLR severity was negatively correlated with mean (r = -0.19, p=0.007), median (r = -0.19, p=0.009), and maximum temperatures (r = -0.16, p=0.02). These negative associations suggest that lower temperatures favor CLR development.

#### 3.9.2 CLR and humidity, leaf wetness, dewpoint, and rainfall

Positive correlations were found between CLR incidence and both mean relative humidity (r = 0.18, p = 0.01) and mean leaf wetness (r = 0.21, p=0.003). In contrast, mean dewpoint (r = -0.23, p< 0.001) and mean rainfall (r = -0.21, p=0.003) were negatively correlated with CLR incidence. Additionally, CLR severity was negatively correlated with minimum relative humidity (r = -0.16, p=0.002) and mean dewpoint (r = -0.23, p=0.001). These results suggest that high humidity and leaf wetness contribute to CLR colonization.

#### 3.9.3 Hyperparasites and temperature

Like CLR, hyperparasites were negatively correlated with temperature: incidence was negatively correlated with mean (r = -0.22, p =0.002), median (r = -0.24, p<0.001), and minimum temperature (r = -0.15, p=0.02), and severity with minimum (r = -0.19, p=0.007) and maximum temperatures (r = -0.16, p=0.02). These results indicate that, like CLR, hyperparasite colonization was highest at low temperatures.

#### 3.9.4 Hyperparasites and humidity, leaf wetness, dewpoint, and rainfall

In contrast to CLR, hyperparasites were negatively correlated with relative humidity. Both incidence and severity were negatively correlated with maximum humidity (incidence: r = -0.16, p=0.002, severity: r = -0.21, p=0.002). Furthermore, severity was also negatively correlated with minimum (r = -0.25, p<0.001) and median humidity (r = -0.19, p=0.007). Incidence was also negatively correlated with dewpoint (r = -0.23, p=0.007). These results indicate that a moderate and consistent humidity is conducive to hyperparasites development, whereas extremes in humidity may hinder their growth.

## 4 Discussion

### 4.1 Seasonal variation in CLR

The initial goal of this study was to compare current levels and seasonal patterns of CLR distribution in Puerto Rico with previous reports, but we have only found one such report. CLR was first found in Puerto Rico in 1989, over thirty years before it colonized Hawaii in 2020. Yet the scientific literature on CLR in Hawaii is more extensive than that in Puerto Rico, where we have found only five peer-reviewed articles: two on microbiota of urediniospores (Vizcaino et al., 1995; James et al., 2016), one on CLR-resistant coffee cultivars (Rodríguez et al., 2020), one on field sampling strategies (Macchiavelli and Rodriguez, 2000), and one that tracked CLR and associated organisms over one year (Hajian-Forooshani et al., 2023), discussed below. This lack of previous data excludes the possibility of detecting long-term changes in the CLR incidence or severity, but it also gives additional importance and novelty to the present study.

There are several reasons why it would be interesting to study change in CLR distribution and dynamics in Puerto Rico over the last thirty-six years:

1. The genetic composition of the coffee population on the Island has changed in ways that have increased resistance. This change was intentional and perhaps also incidental. For example, periodic hurricanes mean that coffee is extensively replanted. After the most recent hurricanes that severely damaged coffee farms, Irma and Maria in 2017, CLR-susceptible cultivars like Caturra and Bourbon were largely replaced with the CLR-resistant cultivars Limaní, Frontón, Marsellesa and Obatã.

Since *Coffea arabica* is propagated by seed and can outcross, and since different cultivars are often grown together, there can be genetic variation in the progeny due to hybridization. In fact, there is an ongoing debate about whether cultivars in Puerto Rico are true to type or so outcrossed that their identities have changed (Singh-Dhaliwal et al., 1963; WCR, 2025). CLR attacks coffee plants from the cotyledon stage, so selective pressure for resistance in seedlings and heritable variation among seedlings may have led to an increase in resistance over time.

2. As far as is known, Puerto Rico has a single race of *H. vastatrix*, Race II, which suggests the possibility of a single introduction in 1989 (Rodríguez et al., 2020). It is assumed there is no sexual stage. An increase in CLR incidence or severity over time, particularly on resistant cultivars, could suggest recombination (either sexual or somatic) was occurring. This would have serious implications for currently CLR-resistant cultivars and might be expected to increase CLR populations over time.

3. As a small island relatively remote from the nearest continent, trophic structures in Puerto Rico are different from those on the continent, and this extends to interactions of CLR with other organisms (Vandermeer et al., 2024). Many islands are famous for coffee production (for example, Bourbon coffee, grown worldwide and a parent of many newer cultivars, is originally from Reunion), but trophic interactions on coffee farms have only been studied in Mexico and Puerto Rico. The trophic structure on a coffee farm includes potential pathogens and herbivores of CLR, and top-down control by such organisms may be more important in an island like Puerto Rico than on the continent (Hajian-Forooshani et al., 2016, 2023). The introduction of CLR probably favored some of these organisms, whose prevalence may have increased as a result. This might be expected to decrease CLR populations over time.

Our data show a significant overall increase in CLR incidence and severity over the three years of the study. It would be both interesting and useful to see if this is a long-term trend, but previous data are not available. Overall levels were like those reported from Hawaii in the first year of the CLR epiphytotic (Aristizábal and Johnson, 2022).

#### 4.1.1 Dry vs. wet season and correlations with environmental variables

Both incidence and severity of CLR increased significantly during the dry season, from December to March, in all three years (**Figures 2A, B**, **3A, B**). This pattern was very similar to previous reports from México (Pale-Ezquivel et al., 2023), Hawaii (Aristizábal and Johnson, 2022), Brazil (Martins et al., 2015), Ethiopia (Daba et al., 2019; Zewdie et al., 2021), Uganda (Liebig et al., 2019), and several other countries (reviewed by (Van Cauwelaert et al., 2023). The pattern was reversed in other areas of Brazil, where incidence is highest in June and July, when rainfall is low (Zambolim, 2016). As in most of these studies, the peak in CLR incidence followed the harvest season (in Puerto Rico from September through January). In a one-year survey of 25 farms in Puerto Rico, CLR incidence varied greatly among farms, but none showed an increase in the dry season (Hajian-Forooshani et al., 2023); however, the proportion of CLR-infected plants was measured instead of the proportion of CLR-infected leaves, so results are not directly comparable. Severity was not reported in that study.

During this study, the dry season was characterized by lower temperatures, with a minimum of 11.8°C, a mean of 21.4°C, and a maximum of 30.9°C. CLR incidence and severity were significantly and negatively correlated with mean, median, and maximum temperature. Similarly, in Brazil, Martins et al. (2015) found a negative relationship between CLR incidence and maximum temperature during the dry season. These results suggest that the lower temperatures occurring during the dry season from December to February favor rust penetration and colonization; according to Kushalappa and Eskes (1989), colonization is more influenced by temperature than leaf wetness.

CLR infection involves several key processes, including uredinospore adhesion to the host surface, germination, appressorium formation, penetration through stomata, and inter- and intracellular colonization (Talhinhas et al., 2017). For germination, optimal temperatures are 21 to 23°C, while penetration through the stomata is stimulated at 17°C and appressorium formation occurs fastest at 13 to 16°C (De Jong et al., 1987; Tadesse et al., 2021; Avelino et al., 2023). After germination and penetration, a latent period follows before leaf colonization begins. This period is shortest at 18 to 28°C (Zambolim, 2016; Avelino et al., 2023). Temperatures below 12.5°C and above 32.5°C prevent spore germination (Kushalappa et al., 1983; De Jong et al., 1987).

CLR incidence was also negatively correlated with mean rainfall, which is consistent with most previous studies on CLR dynamics; heavy rains can wash urediniospores off the leaves and carry them to the ground (Avelino et al., 2020; Sudha et al., 2020; Sera et al., 2022; Avelino et al., 2023). However, rainfall also plays an important role in urediniospore release and movement (Avelino et al., 2023). Low-intensity rainfall events during the dry season probably are sufficient to dislodge rust spores from leaves, but not enough to wash them away. This facilitates initial dispersion, as water droplets can carry the spores to other leaves or nearby plants. As in our results, in Brazil high levels of CLR incidence were observed when the temperatures and rainfall were low (Zambolim, 2016).

CLR incidence was positively correlated with mean relative humidity and leaf wetness. Humidity is a key factor in CLR infection (Kushalappa and Eskes, 1989; Tadesse et al., 2021; Salazar-Navarro et al., 2024), affecting mainly the germination and penetration of urediniospores (Tadesse et al., 2021). However, humidity alone is not enough to complete the process of infection (Yirga, 2020): sustained leaf wetness is necessary for germination (Avelino et al., 2015). In humid, tropical regions like Puerto Rico, leaf wetness remains high even on dry days due to dew; free water on the leaf surface is required to ensure the germination (Kushalappa and Eskes, 1989).

Fruit production and harvesting also have a major influence on CLR, for two reasons: first, a plant that recently invested much of its resources in fruit production may have fewer resources for defense against pathogens (Zambolim, 2016). The effect of fruit load on susceptibility to CLR was even seen in leaf disks *in vitro* (see (Avelino et al., 2023). Also, the harvest requires movement of workers and equipment, and they may transport rust urediniospores (Avelino et al., 2015). Furthermore, leaves are mostly produced at the start of the rainy season, and by the time of the dry season, they have had more time to accumulate pathogen load.

All these factors may still not fully explain seasonal patterns in CLR infection. Management and microclimate may influence variation among plots, and the distance from other coffee plots and the density and type of shade trees affect the movement of spores (Avelino et al., 2023; Van Cauwelaert et al., 2023).

### 4.2 Branch height

CLR incidence and severity were significantly higher on lower and middle branches than on upper branches. In Hawaii and Brazil, CLR incidence was also higher on lower branches, and in Brazil, also in middle branches; differences in severity were not reported in either study (Martins et al., 2015; Aristizábal and Johnson, 2022). Other studies sampled both higher and lower strata but did not report differences (Daba et al., 2019; Belachew et al., 2020).

Exposure to direct sunlight inhibits urediniospore viability and affects infection and colonization (Avelino et al., 2023). Shade provided by the middle and upper branches of the coffee plant may create a microclimate favorable to CLR germination and colonization by reducing sunlight exposure and temperature while maintaining leaf wetness and relative humidity. This may explain the higher incidence and severity observed on branches in the lower canopy, as reported in coffee plots growing under the shade of the leguminous tree *Erythrina poeppigiana* (López-Bravo et al., 2012).

### 4.3 Altitude

Both CLR incidence and severity increased with altitude. These differences were significant even though the altitude range in Puerto Rico is much smaller than in most coffee-growing countries: the highest and lowest sites sampled differed by only 548 m (**Table 1**). Despite the relatively small differences in altitude among the fifteen sampled sites, temperature decreased with increasing altitude (**Table 2**). Correlation analysis showed that several processes involved in CLR development, such as colonization, penetration, and germination were higher at cooler temperatures. In Hawaii, CLR incidence and severity also increased with altitude, at least during harvest season, although the differences were not significant (Aristizábal and Johnson, 2022). In general, the literature says that CLR is less severe at higher altitudes, because diurnal fluctuation in temperatures is greater and temperatures at night are lower (Kushalappa and Eskes, 1989). However, this is based on data from countries where coffee is grown at >1500 m. The highest sites sampled here were<1000 m, so all these studies would support an increase in CLR at intermediate altitudes ~ 1000 m. Interactions of the effects of altitude, climate, shade, and management are complex (Liebig et al., 2019). In Puerto Rico, the higher altitudes that are more conducive to CLR are also more conducive to the coffee berry borer (*Hypothenemus hampei*), another major impediment to coffee cultivation (Mariño et al., 2017). But coffee at lower altitudes is increasingly threatened by climate change (Fain et al., 2018), leaving prospective coffee farmers with a difficult choice.

### 4.4 Differences among *Coffea arabica* cultivars

Of the three resistant cultivars (as classified by World Coffee Research (WCR, 2025)), Obatã and Marsellesa had significantly higher incidence and severity than Limaní. Obatã and Marsellesa were widely planted in Puerto Rico after Hurricanes Irma and Maria in 2017. Limaní is a Sarchimor cultivar that was selected in Puerto Rico. Since genotype is not the sole determinant of susceptibility, it may be than Marsellesa and Obatã are less resistant in Puerto Rico than in Nicaragua and Brazil, where they were selected. Marsellesa was reported to have 0% CLR incidence in Colombia at >1400 m (Dilas-Jiménez et al., 2022). The cultivars rated as ‘low resistance/susceptible’ by WCR also varied significantly in resistance. Caturra was significantly more susceptible than the others.

The most disturbing result was that the ‘highly resistant’ cultivars Obatá, Marsellesa and Limaní had, on average, half the CLR incidence and severity than the ‘low resistance/susceptible’ cultivars. This difference was significant, but it shows that cultivar alone is not a complete protection against CLR. Furthermore, all three resistant cultivars evaluated in this study are sarchimores (crosses between Villa Sarchi and HDT CIFC832/2), with CLR resistance conferred by *C. canephora* genes SH6, SH7, SH8, and SH9 from the Timor Hybrid (Talhinhas et al., 2017; WCR, 2025). Since only race II of CLR [which only infects Arabica coffee (Rodrigues et al., 1975)] has been reported in Puerto Rico (Rodríguez et al., 2020), these findings suggest that additional CLR races could be present.

Apart from genotype, many other factors affect the degree of resistance of coffee cultivars. Yield determined 35% of the variation in susceptibility among accessions in Ethiopia (Eskes and Carvalho, 1983). We did not measure yield. Leaf age is another factor that affects susceptibility (Kushalappa and Eskes, 1989). We did not measure leaf age. Genotype/environment interactions (GxE) affect disease susceptibility (Ngure and Watanabe, 2024).

It is not clear if the variation in resistance observed among cultivars is stable over time or in flux. On the evolution of plant-rust dynamics, most emphasis has been placed on the capacity of the pathogen to evolve new races (McDonald and Linde, 2002). However, as far as is known, only Race II of CLR is present in Puerto Rico (Rodríguez et al., 2020), and there is no evidence for recombination. On the other hand, coffee plants are propagated by seed, and outcrossing among cultivars may occur, as mentioned in 4.1. Comparisons of the susceptibility of widely-planted resistant cultivars like Marsellesa and Obatã over time and among sites would be useful for resolving these issues.

### 4.5 Hyperparasites

Incidence and severity of hyperparasites increased significantly in the dry season, as did that of their CLR host. Similarly, in Brazil, hyperparasitism increased during the dry season (Martins et al., 2015). However, this pattern was not found in a previous study of 25 farms in Puerto Rico, in which incidence of hyperparasites varied greatly among farms and did not track CLR incidence in most of them (Hajian-Forooshani et al., 2023). And in Ethiopia, one study found that hyperparasitism increased during the wet season and another during the dry season (Zewdie et al., 2021; Ayalew et al., 2024).

Both the incidence and severity of hyperparasites were positively correlated with CLR incidence and severity. Since hyperparasite activity depends directly on its host, many environmental factors that affect CLR may also impact its hyperparasites. Like CLR, incidence and severity of hyperparasites were negatively correlated with minimum, mean, median, and maximum temperatures, indicating that their colonization and development are favored at low temperatures. Consistent with our findings, studies in Ethiopia (Ayalew et al., 2024) and in Brazil (Martins et al., 2015) also reported that hyperparasites are more frequent at low temperatures.

Incidence of hyperparasites was negatively correlated with maximum relative humidity and dew point, while severity was negatively correlated with minimum relative humidity. These results suggest that hyperparasites are more sensitive to environmental humidity than CLR. They require moderate and consistent relative humidity to support their colonization and development. Sudden peaks in humidity or excessively dry conditions may hinder their growth or reduce their competitive ability. Low humidity on the leaf surface could be a limiting factor for hyperparasites’ development (Zewdie et al., 2021).

Severity of hyperparasites also increased when shade cover increased, which agrees with our result that incidence and severity of hyperparasites were significantly higher on lower branches than on middle and higher branches. Similarly, in Brazil, hyperparasitism was higher in lower parts of the plant (Martins et al., 2015). Another study found that incidence of hyperparasites was higher and more consistent in Puerto Rico than at a similar latitude in Chiapas, Mexico (Vandermeer et al., 2024).

The significant increase in severity of hyperparasites on CLR-susceptible cultivars vs. CLR-resistant cultivars might not reflect any interaction of the hyperparasites with the plant; the more extensive CLR growth on CLR-susceptible plants may provide the hyperparasites with more substrate to grow on.

The community of CLR hyperparasites in Puerto Rico is diverse, including several species and genera (manuscript in preparation). Those considered here are superficially similar and are collectively referred to in many studies as *Lecanicillium* (or in older literature, *Verticillium* and recently, *Akanthomyces*). These species likely respond to environmental conditions in different ways, and the above analysis oversimplifies complex dynamics among different fungi in a similar niche.

### 4.6 Conclusions and recommendations

There is extensive evidence to support all the factors mentioned in Section 4.1 as determinants of CLR incidence and severity, but economic factors are also involved. Epiphytotic peaks of CLR in Central America and Colombia have coincided with low coffee prices; lack of income may prevent coffee farmers from preventative measures they would have taken had they received higher prices (Avelino et al., 2015).

Do the levels of CLR incidence reported here suggest that rust control strategies should be used? Assuming a management threshold of 5% incidence (Zambolim, 2016), all the fifteen sites we sampled would benefit from control measures, including those with resistant cultivars. This would be a marked change from current practice. The site with the lowest CLR incidence over three years, planted with the resistant cultivar Limaní, had an average 15.2% incidence, three times above the recommended threshold. Yet no CLR control measures were used on almost half of the sites (**Table 1**).

Based on the phenology reported here, preventive methods would be most effective if applied in May and June, when CLR levels typically begin to rise. Management could include the application of mycoparasites like *Lecanicillium* in October and November when they are most likely to survive and multiply. If synthetic fungicides are used, we recommend a considerable window of time between the applications of preventive fungicides and mycoparasites, since fungicides could reduce the survival of hyperparasites.

## Data Availability

The original contributions presented in the study are included in the article/supplementary material. Further inquiries can be directed to the corresponding author.
